# Automatic annotation of eukaryotic genes, pseudogenes and promoters

**DOI:** 10.1186/gb-2006-7-s1-s10

**Published:** 2006-08-07

**Authors:** Victor Solovyev, Peter Kosarev, Igor Seledsov, Denis Vorobyev

**Affiliations:** 1 Department of Computer Science, Royal Holloway, University of London, Egham, Surrey TW20 0EX, UK; 2 Softberry Inc., Radio Circle, Mount Kisco, NY10549, USA

## Abstract

**Background:**

The ENCODE gene prediction workshop (EGASP) has been organized to evaluate how well state-of-the-art automatic gene finding methods are able to reproduce the manual and experimental gene annotation of the human genome. We have used Softberry gene finding software to predict genes, pseudogenes and promoters in 44 selected ENCODE sequences representing approximately 1% (30 Mb) of the human genome. Predictions of gene finding programs were evaluated in terms of their ability to reproduce the ENCODE-HAVANA annotation.

**Results:**

The Fgenesh++ gene prediction pipeline can identify 91% of coding nucleotides with a specificity of 90%. Our automatic pseudogene finder (PSF program) found 90% of the manually annotated pseudogenes and some new ones. The Fprom promoter prediction program identifies 80% of TATA promoters sequences with one false positive prediction per 2,000 base-pairs (bp) and 50% of TATA-less promoters with one false positive prediction per 650 bp. It can be used to identify transcription start sites upstream of annotated coding parts of genes found by gene prediction software.

**Conclusion:**

We review our software and underlying methods for identifying these three important structural and functional genome components and discuss the accuracy of predictions, recent advances and open problems in annotating genomic sequences. We have demonstrated that our methods can be effectively used for initial automatic annotation of the eukaryotic genome.

## Background

The successful completion of the Human Genome Project has demonstrated that large-scale sequencing projects can generate high-quality data at a reasonable cost. In addition to the human genome, researchers have already sequenced the genomes of a number of important model organisms that are commonly used as test beds in studying human biology. These are chimpanzee, mouse, rat, two puffer fish, two fruit flies, two sea squirts, two roundworms, and baker's yeast.

Currently, sequencing centers are close to completing working drafts of the genomes of chicken, dog, honey bee, sea urchin and a set of four fungi, and variety of other genomes are currently in the sequencing pipelines [1].

Many new genomes lack such rich experimental information as the human genome and, therefore, their initial computational annotation is even more important as a starting point for further research to uncover their biology. The more comprehensive and accurate are such computational analyses, the less time-consuming and costly experimental work will have to be done to determine all functional elements in new genomes. Using computational predictions, the scientific community can get at least partial knowledge of a majority of real genes, because gene finding programs usually correctly predict most exons of each gene.

The National Human Genome Research Institute (NHGRI) has initiated the ENCODE project to discover all human genome functional elements [2]. Its pilot phase is focused on performance evaluation of different techniques of genome annotation, including computational analysis, on a specified 30 Mb of human genome sequence. The 2005 ENCODE gene prediction workshop (E-GASP '05) [3] was organized to evaluate how well automatic annotation methods are able to reproduce manual annotations.

This paper describes computational methods for identifying three important structural and functional genome components: genes, pseudogenes and promoters. We used Softberry gene finding software to predict genes, pseudo-genes and promoters in 44 ENCODE sequences. We review the performance of our software and underlying methods for identifying these three important structural and functional genome components, and discuss the accuracy of predictions, recent advances and open problems in annotating genomic sequences.

## Results and discussion

### Running Fgenesh++ on ENCODE sequences

Two sets of ENCODE sequences were prepared to run on the gene prediction pipeline: 44 original ENCODE sequences, and 44 ENCODE sequences with repeats masked by N. Files with coordinates of repeats were downloaded from UCSC web pages devoted to ENCODE project [4]. Low complexity regions and simple repeats were not masked. All three steps of the pipeline were run to annotate ENCODE sequences.

#### Step 1: mapping known mRNAs and selecting good mappings

A set of known human mRNA sequences was prepared from RefSeq. Only RefSeq records with an accession prefix NM_ and a status key REVIEWED, that is, those corresponding to curated and reviewed RefSeq mRNA records, were taken into account. Known mRNAs were mapped by Est_map to 44 ENCODE sequences, and good mappings were automatically selected by the pipeline. Areas corresponding to mapped mRNAs were masked to exclude them from subsequent gene prediction steps.

#### Step 2: mapping known proteins by Prot_map followed by protein homology-based gene prediction by Fgenesh+

In this step, genes are predicted based on homology to known proteins - as a rule, it improves quality of predicted gene models. The NR (non-redundant) database of protein sequences was used as a source of known proteins. First, gene models were predicted using a combination of Prot_map and Fgenesh+: Prot_map maps the NR database to genomic sequences, and Fgenesh+ predicts more refined gene models in regions corresponding to mapped proteins. Then, predicted gene models were additionally filtered by a script that analyses blast2 alignment between predicted proteins and protein homologs. Only reliable models that have a blast score >100 and coverage >80% for both proteins and homologs were selected.

#### Step 3: ab initio gene prediction

In this step, special scripts prepared sequence fragments that contained no gene models from steps 1 and 2. Then gene models in these sequence fragments were predicted *ab initio *by Fgenesh. Finally, gene predictions were converted from the Fgenesh-like output format into GTF format, which is required for submission of results to E-GASP '05.

### Results of Fgenesh++ application to ENCODE sequences

While doing calculations for EGASP, we annotated ENCODE regions of the hg16 version (NCBI build 34). HAVANA annotation, against which results were compared by EGASP, was done on the hg17 version. Four ENCODE sequences were changed upon transition from hg16 to hg17: ENm006, ENm014, ENr131, ENr211. We re-annotated these four sequences (after the EGASP deadline), and the results presented here include this correction.

When calculating the prediction accuracy, only coding sequence (CDS) blocks, from Softberry predictions as well as from the HAVANA annotation, were taken into account. We used the HAVANA annotation file '44regions_coding.gff', the version of 7 June 2005, which describes 1,078 transcripts with CDS containing 673,501 nucleotides (the HAVANA annotation was taken into account only within the range of ENCODE sequences). The accuracy results are presented in Table 1. At the nucleotide level we estimated sensitivity (Sn) as the percentage of true coding bases that were correctly predicted as coding, and specificity (Sp) as the percentage of bases predicted to be in coding regions that were actually coding. We observed Sn = 0.9 and Sp = 0.8 at the nucleotide level. To measure accuracy at the CDS level, a non-redundant set of CDS was considered. Sensitivity (Sn) at the CDS level is the number of CDS predicted correctly divided by the number of known CDS, and specificity (Sp) is the number of predicted CDS that are correct divided by the number of all predicted CDS. When calculating the accuracies, CDS orientation is checked for known and predicted CDS. We observed Sn = 0.78 and Sp = 0.74 at the CDS level. More than 50% of predicted coding bases were predicted with the help of homologous proteins from the NR database, and approximately 35% were predicted with the help of mRNAs from RefSeq.

Performance at the level of exact prediction of all CDS in a gene is presented in Table 2. We can see that all CDSs were predicted exactly for 61% and 52% of genes computed with mRNA and protein support, respectively. It is interesting to note that the Sp for *ab initio *predictions, which comprise approximately 13% of all predicted nucleotides, is very low. If we exclude *ab initio *predictions and calculate an accuracy only for mRNA and protein supported predictions, the specificity rises up to 89.5% at the nucleotide level with just a slight decrease in sensitivity (Table 3). On the other hand, if we run just *ab initio *predictions for 44 ENCODE sequences, we have Sn = 0.88 and Sp = 0.74 at the nucleotide and CDS levels, respectively (Table 3). That is significantly higher than the values for *ab initio *predictions in Table 1. It might indicate that regions having neither known mRNAs nor homology to known proteins can contain genes that are missed in the HAVANA annotation. Another interesting observation is that *ab initio *gene finding demonstrates a good performance at the nucleotide level (Sn = 0.88, Sp = 0.74), while it is relatively weak at the level of exact CDS prediction, compared to mRNA- or protein-supported predictions. *Ab initio *predictions seem to usually contain one or several errors in a set of gene CDSs, as well as tend to split one gene into two or merge neighbor genes more often.

We did not use expressed sequence tag (EST) information [5] in the generation of our predictions that resulted in the smaller number of predicted alternative transcripts compared with the HAVANA annotation. EST data also can be used for extension of terminal coding exons to their 5' or 3' non-coding parts. Including EST data as well as inter-genome similarity data can further improve the annotation quality of our gene prediction pipeline.

### Prediction of pseudogenes

**Figure 1:**
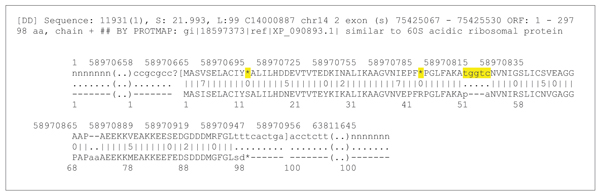
Example of a processed pseudogene. Alignment versus protein encoded by the parent gene. Identity, 83.7%; coverage of protein sequence, 93.9%; number of internal stop codons, 2; number of frameshifts, I; K_a_/K_s_, 0.484.

**Figure 2:**
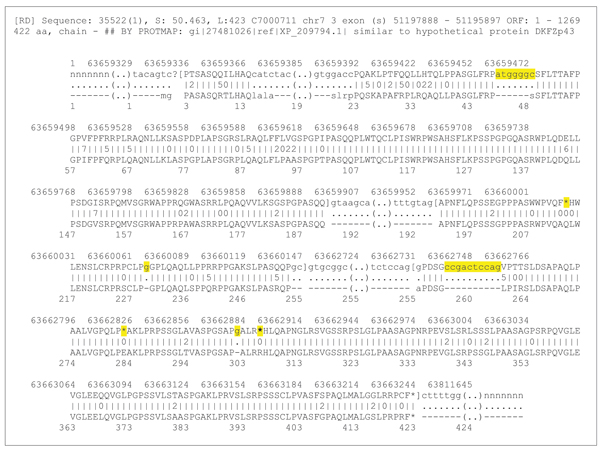
Example of a pseudogene that has not been processed. Alignment versus protein encoded by the parent gene. Identity, 86.4%; coverage of protein sequence, 97.6%; number of internal stop codons, 3; number of frameshifts, 4; K_a_/K_s_, 0.594.

We used Softberry gene PSF (pseudogene finding) to identify pseudogenes in 44 ENCODE sequences. This program, described in Materials and methods, recognizes pseudogene sequences using some characteristics of genome alignment regions with their parent proteins. Examples of two types of pseudogenes, processed and non-processed, and their characteristics are presented in Figures 1 and 2.

We presented to EGASP two sets of pseudogenes found in ENCODE sequences (hg16 release). Four ENCODE sequences were changed upon transition from hg16 to hg17 (ENm006, ENm014, ENr131, ENr211) and the results presented here exclude them. One set, which we called 'reliable set', contained 56 processed pseudogenes, 93% of which almost completely overlap with 52 of 145 HAVANA pseudogenes. Overall, 80 (59%) of 135 pseudogenes from two sets overlapped 82 (57%) of 145 HAVANA pseudogenes.

We improved our PSF automatic pseudogene predictor and reran it. As a result, we found 181 potential pseudogenes, 118 of which had a significant overlap with the annotated 145 HAVANA pseudogenes. Of these 118 pseudogenes, 68 (58%) had only one exon and could be classified as processed pseudogenes: 58 had the parent gene with more than one exon and 7 others had polyA tail. Of the 118 pseudogenes, 106 (90%) had one or more defects in their open reading frames (ORFs). Among the remaining 12, there are 4 pseudogenes with a single exon (while their parents have 4 or more exons), 4 contain both polyA signal and polyA tract, 4 have only a polyA tract, and 2 have only high Ka/Ks ratios (0.59 and 1.04).

**Figure 3:**
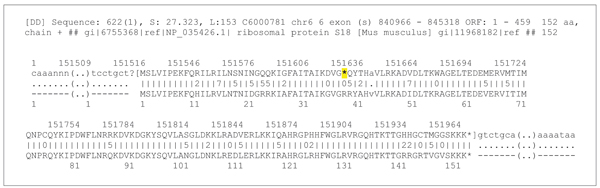
Pseudogene in ENm004 sequence, absent from HAVANA annotation. The alignment has a stop codon close to position 151636.

PSF did not find 27 HAVANA annotated pseudogenes. Three of them were not reported because they are located in introns of larger pseudogenes (AC006326.4-001, AC006326.2-001 and AL162151.3-001). The other 10 represent fragments of some human proteins and are missing stop codons or frameshifts. We did not include pseudogenes corresponding to fragments of proteins in our pseudogene set. The remaining 14 HAVANA pseudogenes were not found, probably because of some limitation of our program and the processed datasets. Some of them might have parent genes that were absent from our initial protein set compiled by the Fgenesh++ gene prediction pipeline. Some of the 63 pseudogenes that have been predicted by PSF but were absent from the HAVANA set might have appeared because of imperfect predictions by the pipeline, which produced frameshifts when a pseudogene candidate and its parent gene were aligned. However, some of these 'over-predicted' pseudogenes might be actual pseudogenes missed by the HAVANA annotators (see Figure 3 for such an example).

To summarize, the PSF pseudogene prediction program found 81% of annotated pseudogenes. Its quality can further be improved by improving the quality of parent gene-protein sets.

### Pol-II promoter recognition

Since each eukaryotic polymerase II promoter has a unique selection and arrangement of regulatory elements, which provide unique instructions for gene expression, the computational identification of promoters in genomic DNA is an extremely difficult problem [6]. This task is two-fold: finding the exact position of a transcription start site within a long upstream region of a typical eukaryotic gene; and avoiding false positive predictions within exon and intron sequences. To resolve the second problem, some authors of promoter finding software include special procedures for recognition of coding parts of gene blocks inside promoter prediction programs [7,8]. However, gene prediction software such as Genscan [9] or Fgenesh [6,10] provides much better accuracy in the identification of coding exons and introns than any such procedures. We think that the best promoter identification strategy is to combine prediction of all gene components in one program. While trying to create such a program, we decided to use some intermediate variant that includes the following steps: computation of gene annotation using a gene prediction pipeline, and promoter prediction within 5' regions upstream of the annotated coding regions of predicted genes.

**Figure 4:**
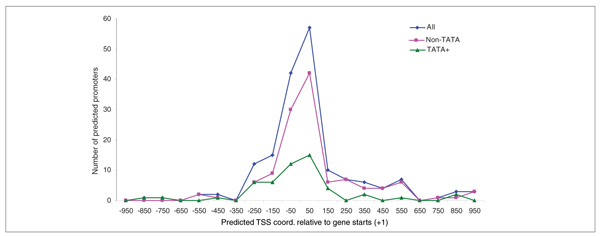
A distribution of predicted TSS relative to the start of mRNA sequences. Figures on the x-axis are centers of 100 bp intervals, for example, mark 50 corresponds to [+1,+100] interval.

We extracted 5' regions (upstream from the first CDS) from predicted genes and ran Fprom on these sequences. For each region, we selected one predicted promoter closest to the CDS and presented it in our results. There are no data on the exact location of transcription start sites for most of genes. But 5' ends of 'full length' mRNAs from Refseq could, on average, be considered pretty close to actual transcription start sites, whereas their 3' ends are often incomplete. With this in mind, we estimated the accuracy of promoter prediction on 251 genes derived from known Refseq mRNAs with >40 bp in their 5' non-coding sequence. Promoters were predicted for 90% (226) of them. Among them, there were 95 TATA+ and 131 TATA- promoters. Figure 4 shows how close predicted promoters are to starts of corresponding mRNAs.

**Figure 5:**
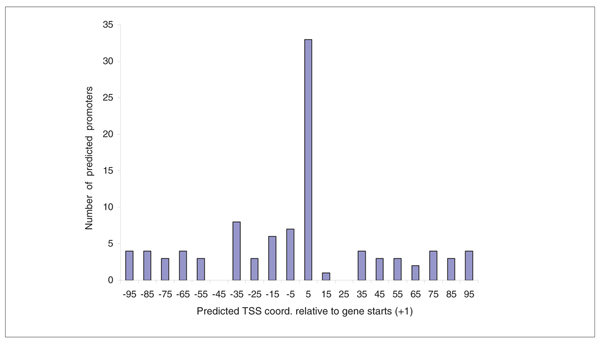
A distribution of predicted TSS near the start of mRNA sequences. Figures on the x-axis are centers of 10 bp intervals, for example, mark 5 corresponds to [+1,+10] interval.

In Figure 5, we see a sharp peak showing that a substantial fraction of predicted promoters is as close as several bases to mRNA starts. The ability to find many promoters with such precision is a remarkable characteristic of our program that distinguishes it from many promoter finding programs that usually assign promoter within a 200 to 1,000 bp range around actual Transcription Start Site (TSS). We should take into account the occurrence of multiple transcription start sites, especially in genes with TATA-less promoters; therefore, some scattering of the predictions around the annotated TSS should not be unusual. Some predictions that deviate significantly from known 5' ends of mRNAs could belong to alternative promoters, which are not unusual for human genes. The histogram (Figure 5) might serve as an approximate criterion of program quality and can be used to compare results produced by different approaches. The ideal test should be done with experimentally verified transcription start sites, but accounting for multiple TSSs will present complications even in such a setting.

## Conclusion

In this paper we present an implementation of three computational pipelines (Fgenesh++, PSF and Fprom) for automatic identification of protein coding genes, pseudo-genes and promoters in eukaryotic genomes. These pipelines, applied to analysis of 44 selected ENCODE sequences, demonstrated an ability to reproduce, to a significant extent, the manual ENCODE-HAVANA annotation. Fgenesh++ gene prediction pipeline can identify 91% of coding nucleotides with a specificity of 90%. The automatic pseudogene finder (PSF program) found 90% of manually annotated pseudogenes and some new ones. Fprom promoter prediction program identifies 80% of TATA promoter sequences with one false positive prediction per 2,000 base pairs (bp), and 50% of TATA-less promoters with one false positive per 650 bp. It can be used to identify transcription start sites upstream of annotated coding parts of genes found by gene prediction software. Thus, the pipelines could be used for easy and fast production of reasonably accurate first pass annotation of a new genome. The described software and its components can be run on computers with Unix operation systems, as well as with Windows as part of the Molquest program package.

## Materials and methods

### Fgenesh++ gene identification pipeline

About 41% of sequenced human DNA consists of different kinds of repeats. Only approximately 3% of the genome sequence contains protein coding exon sequences. Gene sizes can be as large as hundreds of megabases in vertebrates, especially in primates. The average size of an exon is about 190 bp, which is close to the DNA length associated with a nucleosome particle. Human exons are significantly smaller than genes. There are many exons as short as several bases. Moreover, the same DNA sequences may code several different proteins due to alternative promoters or terminators and alternative splicing. These processes make computational gene finding a rather nontrivial task.

#### Hidden Markov model based eukaryotic gene identification

Exons, introns, 5' and 3' UTRs regions are different components (states) of gene structure that occupy k non-overlapping subsequences of a sequence. There are 35 states in a eukaryotic gene model, considering direct and reverse chains as possible gene location. A gene structure can be considered as an ordered set of state/sub-sequence pairs, φ = {(q_1_, x_1_), (q_2_, x_2_), ... ,(q_k_, x_k_)}, called a parse. A parse φ is considered a predicted gene structure if probability *P*(*X*, *φ*) of generating X according to φ is maximal over all possible parses, or when a score is optimal in some meaningful sense. This probability can be computed using statistical parameters describing a particular state and generated from a training set of known gene structures and sequences. Successive states of this hidden Markov model (HMM) are generated according to the Markov process with inclusion of explicit state duration density. A simple technique based on a dynamic programming method for finding an optimal parse, or the best sequence of states, is the Viterbi algorithm, which requires o(N^2^D^2^L) calculations, where N is the number of states, D is the longest duration and L is the sequence length [11]. A helpful technique to reduce the number of states and simplify computations by modeling non-coding state length with a geometrical distribution to predict multiple genes was initially implemented in the Genscan algorithm [9]. Several other successful HMM-based gene finding programs, such as HMMgene [12], a variant of Genie [13] and GeneMark [14], and Fgenesh [6,10] have been developed. Fgenesh (Find GENES using Hmm) is currently one of the most accurate and the fastest program. The run time of Fgenesh is practically linear, and the current version has no practical limit on length of analyzed sequence. Predicting genes in 34.5 Mb of human chromosome 22 sequence takes about 1.5 minutes with a EV6 Decalpha processor and is even faster on modern Linux computers.

An *ab initio *gene prediction program such as Fgenesh predicts about 93% of all coding exon bases and exactly predicts about 80% of human exons when applied to single gene sequences (Table 4). Analysis of multi-gene, long genomic sequences is a more complicated task. A program can erroneously join neighboring genes or split a gene into two or more. To improve automatic annotation accuracy, we developed a pipeline Fgenesh++, which can take into account available supporting data such as mRNA or homologous protein sequences.

#### Components of the Fgenesh++ gene prediction pipeline

Fgenesh++ is a pipeline for automatic prediction of genes in eukaryotic genomes without human modification of results. It uses the following sequence analysis software.

#### Fgenesh

Fgenesh is a HMM-based *ab initio *gene prediction program.

#### Fgenesh+

Fgenesh+ is a gene prediction program that uses homologous protein sequence to improve performance.

#### Est_map

Est_map is a program for mapping known mRNAs/ESTs to a genome, producing genome alignment with splice site identification.

#### Prot_map

Prot_map is a program for mapping a protein database to genomic sequence.

#### Est_map

Est_map can map a set of mRNAs/ESTs to a chromosome sequence. For example, 11,000 full-length mRNA sequences from a NCBI reference set were mapped to a 52 Mb unmasked Y chromosome fragment in approximately 20 minutes. Est_map takes into account statistical features of splice sites for more accurate mapping.

#### Prot_map

The Prot_map program maps a set of protein sequences to a genomic sequence, producing gene structures and corresponding alignments of coding exons with similar or identical protein queries. Prot_map uses a genomic sequence and a set of protein sequences as its input data, and reconstructs gene structure based on protein identity or homology, in contrast to a set of unordered alignment fragments generated by Blast [15]. The program is very fast (Table 4), produces gene structures with similar accuracy to those of the relatively slow GeneWise program [16] and does not require knowledge of protein genomic location. The accuracy of gene reconstruction can further be significantly improved using the Fgenesh+ program on the output of Prot_map, that is, a fragment of genomic sequence and the protein sequence mapped to it.

Comparison of accuracy of gene prediction by *ab initio *Fgenesh and gene prediction with protein support by Fgenesh+ or GeneWise and Prot_map was performed on a large set of human genes with homologous proteins from mouse or *Drosophila*. We can see that Fgenesh+ shows the best performance with mouse proteins (Table 5). With *Drosophila *proteins, *ab initio *prediction by Fgenesh works better than GeneWise for all ranges of similarity, and Fgenesh+ is the best predictor if similarity is higher than 60% (Table 6).

Besides the programs listed above, the Fgenesh++ package also includes files with gene finding parameters for specific genomes, configuration files for programs and a number of Perl scripts. In addition, the Fgenesh++ package uses the following public software and data: BLAST executables blastall and bl2seq [15], the NCBI NR database (non-redundant protein database) formatted for BLAST, and the NCBI RefSeq database [17].

Fgenesh++ requires genome sequences and, optionally, the same sequences with repeats masked by N. Sequences can be either complete chromosomes or their fragments, such as scaffolds, contigs, and so on. When preparing repeat-masked sequences, we recommend not masking low complexity regions and simple repeats, as they can be parts of coding sequences.

#### Three main steps of the Fgenesh++ pipeline

There are three main steps in running the pipeline: step 1 involves mapping known mRNAs/cDNAs (for example, from RefSeq) to genomic sequences; step 2 involves the prediction of genes based on homology to known proteins (for example, from NR); and step 3 involves *ab initio *gene prediction in regions having neither mapped mRNAs nor genes predicted based on protein homology.

A user can skip some steps while running the pipeline. For example, to take a first very cursory look at gene models, a user can skip the first two steps and go right to *ab initio *gene predictions. Generally, step 1 (mapping known mRNAs) can be skipped in the following cases: if there is no representative collection of known mRNAs for a query genome, that is, RefSeq does not contain enough entries and the user does not have their own collection; and if genomic sequences are fragmented, so that individual mRNAs are likely to be broken among several genomic fragments. The output of the pipeline consists of predicted gene structures and corresponding proteins. It also indicates whether particular gene structure was assigned based on mRNA mapping, protein homology, or *ab initio *gene prediction.

### The pseudogene annotation program (PSF)

Our method of searching for pseudogenes can work with two types of initial information available. One type contains exon-intron structures of annotated genes and their protein sequences for a genome under analysis. To get such information, we can execute a gene finding pipeline, such as Fgenesh++. In this case, we run Prot_map program with a set of protein sequences to find possible significant genome-protein alignments that do not correspond to a location of a gene for mapped protein. Another type of initial data can be a set of known proteins for a given organism. Having such data, we can restore gene structure of a given protein using the Prot_map program. For each mapped protein, we can select the best scoring mapping and the computed exon-intron structure as the 'parent' gene structure of this protein. If the alignment of a protein with its own parent has obvious internal stop codons or frameshifts, this locus could be included in the list of potential pseudogenes, but we need to keep in mind more trivial explanations, such as sequencing errors. Such loci cannot be analyzed on the basis of their Ka/Ks or checked for intron losses. In any case, for each of two cases we have a set of protein sequences, their parent gene structures, and protein-genome alignments for further analysis to identify pseudogenes.

#### Selecting potential pseudogenes

Using genome-protein alignments generated by the Prot_map program, the PSF program produces a list of alignments possessing the following properties for each protein. First, the identity in blocks of alignment exceeds a certain value. Second, a substantial portion of protein sequence is included in the alignment. Third, the genomic location of alignment differs from that of parent gene. And fourth, at least one of four events is observed: damage to an ORF - there is one or more frameshifts or internal stop codons; a single exon with a close poly-A site - the poly-A site is too close to a 3' end of an alignment, while the carboxyl terminus of the protein sequence is aligned to the last amino acid, and a single exon covers 95% of protein sequence; loss of introns - protein coverage by alignment is at least 95%, and the number of exons is fewer than in the parent gene by a certain number; or the protein sequence is not preserved - the ratio of non-synonymous to synonymous replacements exceeds a certain threshold (Ka/Ks > 0.5). Ka/Ks is calculated relative to the parent gene by the method presented by Nei and Gojobori [18].

#### Selecting a reliable part of alignment

The procedures described apply to a so-called reliable part of alignment. The necessity of introducing this concept appears due to imperfections in aligning a protein against a chromosome sequence. There are complex cases where accurate alignment cannot be produced, such as very short (1 to 3 bp) exons separated by a large intron, or because of some errors in the protein or genome draft sequence that prevent perfect alignment. For instance, if a protein as a whole is well aligned to a chromosome, but about 20 amino acids on its 5' end cannot be aligned in one continuous block, Prot_map will most likely try to align these 20 amino acids by scattering them along several short blocks. Most likely, these blocks will not have any relation to a gene or a pseudogene. Therefore, when searching for pseudogenes, we remove short insignificant trailer blocks. The rest of the alignment is considered its reliable part. To find the reliable part of an alignment, we evaluate the quality of the alignment blocks (exons). For each exon found by Prot_map, we calculate the number of aligned amino acids (M), the number of non-aligned amino acids (AI) and nucleotides (NI) within an exon, and the number of aligned amino acids (AO) and nucleotides (NO) located outside of the exon region to the left and to the right side of an exon. Also, we compute the 'correctness' of splice site conserved dinucleotides (SSC) that flank an exon. If an exon is an amino or carboxy-terminal one, we also compute the 'correctness' of corresponding start or stop codons. The length of an intron (IL) that separates an exon from its nearest exon in the direction of the longest mapped exon is also computed. The empirical 'quality' measure is defined by the following formula:

Q = M - P_AI_(AI) - P_NI_(NI) - P_AO_(AO) - P_NO_(NO) + B_SSC_(SSC) - P_IL_(IL)

where P_AI_, P_NI_, P_AO _and P_NO _are the penalties for the internal and external unaligned amino acids and nucleotides, B_SSC _is a bonus for the correctness of splice sites or start/stop codons, and P_IL _is the penalty for high intron length. The reliable part of the alignment consists of a set of neighboring alignment exons that each have Q > 5.

After Prot_map mapping, many loci on a chromosome include alignments with more than one protein. In such cases, we choose only one most reliable alignment, based on a sum of included exon's qualities.

### FPROM Pol-II promoter recognition program

The gene annotation pipeline was described above. Here we present our promoter recognition program Fprom (find promoter), which is based on further development of an algorithm realized earlier in the TSSW/TSSG programs [6,19]. It was assumed that TATA+ and TATA- promoters have very different sequence features, so these groups were analyzed separately. Potential TATA+ promoter sequences were selected according to the score value of a Bucher TATA box weight matrix [20], with the threshold close to the minimal score value for the TATA+ promoters in the learning set. Selected significant characteristics of the TATA+ promoter group found by discriminant analysis are presented in Table 7.

For each position on a given sequence, the Fprom program evaluates the occurrence of TSS using two linear discriminant functions (separate for TATA+ and TATA- promoters) with characteristics computed in the [-200, +50] region around a given position. If it finds a TATA-box (using a TATA-box weight matrix) in the region, then it computes the value of Linear Discriminant Function (LDF) for TATA+promoters, otherwise the value of LDF for TATA-less promoters. Only one prediction with the highest LDF score and that is greater then a certain threshold is selected within any 300 bp region.

Examples of Fprom predictions are presented in Table 8. The distances between true TSSs and correctly predicted ones varied from matching exactly to 151 bp. It should be noted that experimental mapping of TSSs has the estimated precision of ± 5 bp [20].

Testing Fprom on a control set of 366 TATA and 650 TATA-less promoter sequences demonstrated that the program identified 80% of TATA promoter sequences, with one false positive prediction per 2,000 bp, and 50% of TATA-less promoters, with one false positive prediction per 650 bp. The prediction algorithm described above uses the propensities of each Transcription Factor (TF) binding site [21] independently, not taking into account their mutual orientation and positioning. At the same time, it is well known that transcription regulation is a highly cooperative process, involving simultaneous binding of several transcription factors to their corresponding sites. In future algorithms, we should analyze patterns of regulatory sequences where mutual orientation and location of individual regulatory elements are necessary requirements for their function.

Prediction of genes, ORFs, promoters, and splice sites using the methods described above is available via the web. Fgenesh (*ab initio *gene finding program with parameters for 27 organisms), Fgenesh-M (program for prediction of alternative spliced gene variants), Fgenesh+ (gene prediction based on protein homology), Fgenesh_c (gene prediction with EST support), and Fgenesh2 (gene prediction with support of second, homologous genome sequence) can be found at [22]. Prot_map and Est_map (mapping protein or mRNA/EST, correspondingly, to a genome with exon-intron gene structure reconstruction) is available at [23]. Finding promoter sequences and transcription start sites by Fprom can be executed at [24]. Pseudogene finding software (PSF) is available as a part of Windows-based Molquest package [25] that includes more than a hundred sophisticated sequence analysis programs, including several pipelines and complex visualization components for computational work with biomedical data.

## Figures and Tables

**Table 1:** Accuracy of coding exon prediction by Fgenesh++ pipeline

	All genes, Sn/Sp (%)	mRNA supported, Sp (%)	Protein supported, Sp (%)	*Ab initio*, Sp (%)
Nucleotide level	93.00/79.54	94.19	86.64	13.98
CDS EXACT	78.42/74.18	90.20	84.28	7.85
CDS 1EDGE	91.55/78.51	95.00	88.77	10.47
CDS OVERLAP	92.31/78.85	95.53	89.02	10.74

CDS is considered to be predicted correctly if: both CDS coordinates are predicted correctly (CDS EXACT); at least one CDS edge is predicted correctly (CDS 1EDGE);or predicted CDS overlaps with known CDS (CDS OVERLAP).

**Table 2:** Exact prediction of all CDS in a gene

	All genes	mRNA supported	Protein supported	*Ab initio*
No. of nucleotides predicted (%)	787,505 (100%)	274,889 (34.91%)	417,202 (52.98%)	104,003 (13.21%)
No. of transcripts predicted (%)	820 (100%)	314 (38.29%)	298 (36.34%)	208 (25.37%)
No. of SoftBerry transcripts identical to HAVANA transcripts (%)*	346 of 820 (42%)	191 of 314 (61%)	154 of 298 (52%)	1 of 208 (0.48%)

*Transcripts identical means that their CDS parts (including protein coding exons and coding parts of 5' and 3' exons) are identical; the percentage of SoftBerry transcripts is relative to the number of Softberry transcripts predicted in the corresponding category: mRNA supported, protein supported or *ab initio*.

**Table 3:** Performance data for annotating 44 ENCODE sequences by either mRNA and protein supported or *ab initio *predictions

	mRNA + protein supported, Sn/Sp (%)	*Ab initio*, Sn/Sp (%)
Nucleotide level	91.14/89.54	88.44/74.46
CDS EXACT	77.19/86.48	67.54/64.22
CDS OVERLAP	90.60/91.4	85.00/71.71
SoftBerry transcripts identical to HAVANA transcripts*	56.37% (of 612)	14.75% (of 590)

*Transcripts identical means that their CDS parts (including protein coding exons and coding parts of 5' and 3' exons) are identical; the percentage of SoftBerry transcripts is relative to the number of Softberry transcripts predicted in the corresponding category: mRNA supported, protein supported or *ab initio*.

**Table 4:** Speed of gene prediction programs

	Fgenesh+	Prot_map	GeneWise
88 sequences of genes <20 kb	1 minute	1 minute	90 minutes
8 sequences of genes >400 kb	1 minute	1 minute	1,200 minutes

All values are approximate.

**Table 5:** Accuracy of human gene prediction using similar mouse proteins

	Sn ex	Sno ex	Sp ex	Sn nuc	Sp nuc	CC	%CG
Similarity of mouse protein >90% in 921 sequences							
Fgenesh	86.2	91.7	88.6	93.9	93.4	0.9334	34
Genwise	93.9	97.6	95.9	99.0	99.6	0.9926	66
Fgenesh+	97.3	98.9	98.0	99.1	99.6	0.9936	81
Prot_map	95.9	98.3	96.9	99.1	99.5	0.9924	73
80% < similarity of mouse protein <90% in 1,441 sequences							
Fgenesh	85.8	92.1	87.7	94.0	93.4	0.9334	30
Genewise	92.6	98.0	94.1	98.9	99.5	0.9912	58
Fgenesh+	96.8	99.0	97.2	99.1	99.5	0.9929	77
Prot_map	93.9	98.5	94.1	98.9	99.3	0.9898	60

Sn_ex, sensitivity on the exon level (exact exon predictions); Sno_ex, sensitivity with exon overlap; Sp_ex, specificity on the exon level; Sn_nuc, sensitivity, nucleotides; Sp_nuc, specificity, nucleotides; CC, correlation coefficient; %CG, percent of genes predicted completely correctly (no missing and no extra exons, and all exon boundaries are predicted exactly correctly).

**Table 6:** Accuracy of gene prediction using similar *Drosophila *proteins

	Sn ex	Sno ex	Sp ex	Sn nuc	Sp nuc	CC	%CG
Similarity of *Drosophila *protein >80% in 66 sequences							
Fgenesh	90.5	93.8	95.1	97.9	96.9	0.950	55
Genewise	79.3	83.9	86.8	97.3	99.5	0.985	23
Fgenesh+	95.1	97.8	97.0	98.9	99.5	0.9914	70
Prot_map	86.4	95.3	88.1	97.6	99.0	0.982	41
60% < similarity of *Drosophila *protein <80% in 290 sequences							
*Fgenesh*	88.6	93.1	90.8	94.9	93.8	0.941	34
Genewise	76.3	91.8	82.9	92.8	99.4	0.959	7
Fgenesh+	89.2	94.4	92.7	95.5	98.5	0.968	44
Prot_map	75.1	92.5	74.9	91.4	97.5	0.941	10

**Table 7:** Characteristics of promoter sequences used by Fprom for identification of TATA+ promoters

Characteristics	D^2 ^for TATA+ promoters
Hexaplets in region [-200, -45]	3.1
Hexaplets in region [1, 40]	4.0
TATA box score in region [-45, -25]	2.3
TATA box average score in region [-45, -25]	2.2
Triplets in region [-200, -45]	2.2
Triplets in region [0, 40]	2.9
Position triplet matrix in region [-50, +30]	7.0
Protein-induced deformability	2.9
CpG content	3.0
Similarity in region [-200, -100]	1.0
Motif density in region [-200, -100]	4.5
Protein-DNA-twist	0.3
Motif density in region [-100, -1] (reverse chain)	2.3
Total Mahalonobis distance	14.8
Number of promoters/non-promoters	366/18600

D^2 ^is the Mahalonobis distance [26] showing the strength of characteristics to separate promoter from non-promoter test set sequences.

**Table 8:** Fprom predictions on some GenBank entries with experimentally verified TSS

Gene	GenBank accession number	Length (bp)	True TSS	Predicted TSS	Number of false positives
*CXCR4*	AJ224869	8,747	2,632	2,632	7
*HOX3D*	X61755	4,968	2,280	2,223	7
*DAF*	M64356	2,003	733	795	2
*GJB1*	L47127	950	404	415	2
*ID4*	AF030295	1,473	1,066	1,095	2
*C inhibitor*	M68516	15,571	2,200	2,049	2
*MBD1*	AJ132338	2,951	1,964	1,891	1
*Id -3*	X73428	2,481	665	637	4

GenBank entries are from [27].
